# In(Ga)N 3D Growth on GaN-Buffered On-Axis and Off-Axis (0001) Sapphire Substrates by MOCVD

**DOI:** 10.3390/nano12193496

**Published:** 2022-10-06

**Authors:** Alica Rosová, Edmund Dobročka, Peter Eliáš, Stanislav Hasenöhrl, Michal Kučera, Filip Gucmann, Ján Kuzmík

**Affiliations:** Institute of Electrical Engineering, Slovak Academy of Sciences, Dúbravská Cesta 9, 841 04 Bratislava, Slovakia

**Keywords:** InN, InGaN, quantum dots, epitaxial growth, self-assembly, III-N semiconductors, photoluminescence, TEM

## Abstract

In(Ga)N epitaxial layers were grown on on-axis and off-axis (0001) sapphire substrates with an about 1100 nm-thick GaN buffer layer stack using organometallic chemical vapor deposition at 600 °C. The In(Ga)N layers consisted of a thin (~10–25 nm) continuous layer of small conical pyramids in which large conical pyramids with an approximate height of 50–80 nm were randomly distributed. The large pyramids were grown above the edge-type dislocations which originated in the GaN buffer; the dislocations did not penetrate the large, isolated pyramids. The large pyramids were well crystallized and relaxed with a small quantity of defects, such as dislocations, preferentially located at the contact zones of adjacent pyramids. The low temperature (6.5 K) photoluminescence spectra showed one clear maximum at 853 meV with a full width at half maximum (FWHM) of 75 meV and 859 meV with a FWHM of 80 meV for the off-axis and on-axis samples, respectively.

## 1. Introduction

Recent advances in the epitaxial growth of indium nitride layers led to the correction of the optical InN bandgap to 0.65 eV (previously expected to be 1.9 eV) [1,2] and opened new horizons for InN-based photonic and electronic devices. They include light-emitting diodes (LEDs) and optical cells capable of light emission in the whole visible spectrum, InN-based lasers for 1.55 µm fiber optics, devices for THz spectroscopy, and high-speed transistors [3,4]. New frontiers were also opened for InN-based nanostructures, e.g., for quantum computing, where quantum dot (QD) single photon emitters are required [5].

The most often used techniques to deposit InN or In_1-x_Ga_x_N were the chemical vapor deposition using organometallic precursors (MOCVD or MOVPE) [6,7,8,9,10,11] or molecular beam epitaxy (MBE) [12,13,14]. A significant disadvantage of InN is a necessity to use relatively low deposition temperatures (because of increased InN sublimation at higher temperatures) and lack of native substrates. The large misfit to the typically used GaN of about 11% complicates the deposition of continuous InN layers. Deposition at low temperatures leaded to continuous layers with high densities of threading dislocations and other defects in the InN layers were typically produced [12,15,16]. High temperature deposition of InN resulted in 3D growth of islands or dots that had various shapes and dimensions and were well crystallized with only a few threading dislocations [7,12]. Due to the large misfit between InN and GaN the compressive strain during an initial growth of InN caused that the growing layer reached the critical thickness for island formation very soon (after few monolayers deposition) and the islanding occurred to reduce the strain energy in the Stranski–Krastanov growth mode. After the islanding, when the delivery of the growth species into the reactor continues some of the small islands or their clusters can act as a nucleus site for the preferential large island growth by homoepitaxy and self-assembling of growing islands appears. Using higher deposition temperatures or ammonia stabilisation of InN sublimation some authors confirm 3D Volmer–Weber growth mode from the beginning of the process [12,17].

In this paper we studied the growth of self-assembled InN nano-sized pyramids on GaN-buffered sapphire substrates with two different surface inclinations.

## 2. Materials and Methods

Two samples were prepared in a single growth run on 2-inch c-plane (0001) sapphire substrates using an AIXTRON low-pressure close-coupled showerhead (CCS) organometallic chemical vapor deposition (MOCVD) system. One (off-axis) sample was prepared on a substrate with its surface plane misoriented by 4° from the c-plane toward the ($11\bar{2}0$) a-plane. Such substrates are usually used to improve the quality of GaN growth. The other (on-axis) sample was grown on a c-oriented sapphire substrate without any intentional off-cut.

At first a GaN layer stack was grown as the platform for the subsequent growth of InN. Such GaN stacks are commonly used as the basis for various III-N device structures where a good crystalline quality and surface flatness is needed. It consisted of a low-temperature GaN nucleation layer, a 1000 nm thick GaN coalescence and buffer layer, and a 100 nm thick undoped GaN layer. The stack was deposited from NH_3_ and trimethylgallium (TMGa) in an H_2_ reactor environment. The growth parameters of the thick buffer layer were optimized to achieve high electrical resistance by compensating native n-type defect levels that originate in nitride crystals from carbon doping due to the thermal decomposition of TMGa molecules in the reactor [18,19]. The thin top GaN layer was left undoped to secure a high crystalline quality and high charge carrier mobility. The top GaN layer was grown at the reactor pressure of 400 mbar, deposition temperature of 1080–1090 °C with the V/III ratio of 1400.

After the top GaN layer deposition the growth was interrupted for 470 s and the reactor parameters were set for the InN layer growth. InN was grown from NH_3_ and trimethylindium (TMIn) as the respective N and In precursors using N_2_ as the carrier and purge gas. The reactor pressure was set to 800 mbar, temperature to 600 °C, and growth time to 1295 s. The V/III ratio was 47,000. The growth parameters were optimized to obtain high crystalline quality InN, avoid In droplet formation (occurring at lower growth temperatures), and prevent InN thermal decomposition (occurring at higher growth temperatures) [6].

X-ray diffraction (XRD) experiments were carried out using a Bruker D8 DISCOVER diffractometer equipped with an X-ray source with a rotating Cu anode operating at 12 kW. The measurement was performed in a high-resolution setup with a parabolic Goebel mirror and four-bounce Ge 022 Bartels monochromator in the primary beam. TEM observations were performed using a JEOL JEM 1200EX electron microscope on a cross-sectional TEM specimen prepared with a standard focused ion beam (FIB) milling technique. SEM observations were done with a Quanta FEG-250 environmental scanning electron microscope (SEM). The surface morphology and surface roughness of the samples were acquired using an NT-MDT Ntegra Prima atomic force microscope (AFM) in tapping mode.

Photoluminescence (PL) measurement was realized in a liquid-helium-filled optical cryostat. The samples were pumped using an Argon-ion laser (*λ* = 488 nm). PL radiation was dispersed by a quarter-meter monochromator and detected by an LN_2_-cooled, enhanced-range-InGaAs photodiode. Detector signal was amplified using a standard lock-in technique.

## 3. Results

**Figure 1:**
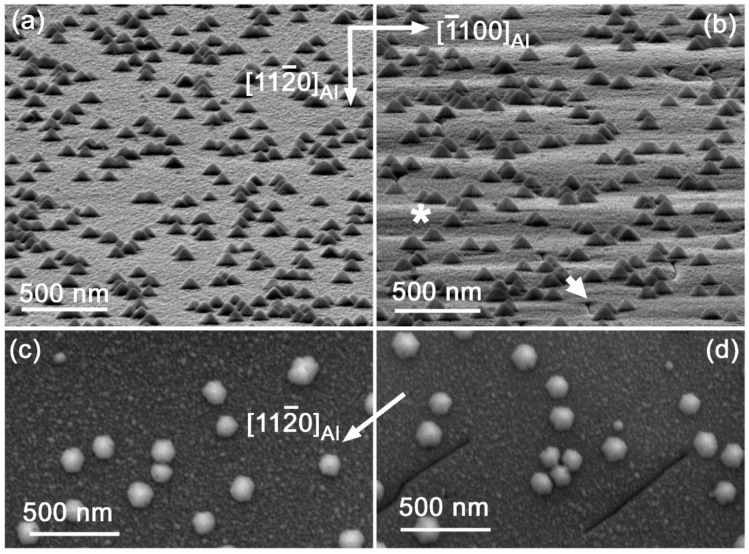
SEM images of the In(Ga)N layers grown on the on-axis (**a**,**c**) and off-axis (**b**,**d**) GaN/Al_2_O_3_ substrates. (**a**,**b**) Images were taken with sample tilted by 85° from the surface normal; (**c**,**d**) are the plane-view images oriented according to the shown direction of Al_2_O_3_ substrate. The asterisk * in (**b**) denotes clearly visible steps related to the off-axis substrate; the thick short arrow points to one of the observed cracks.

**Figure 2:**
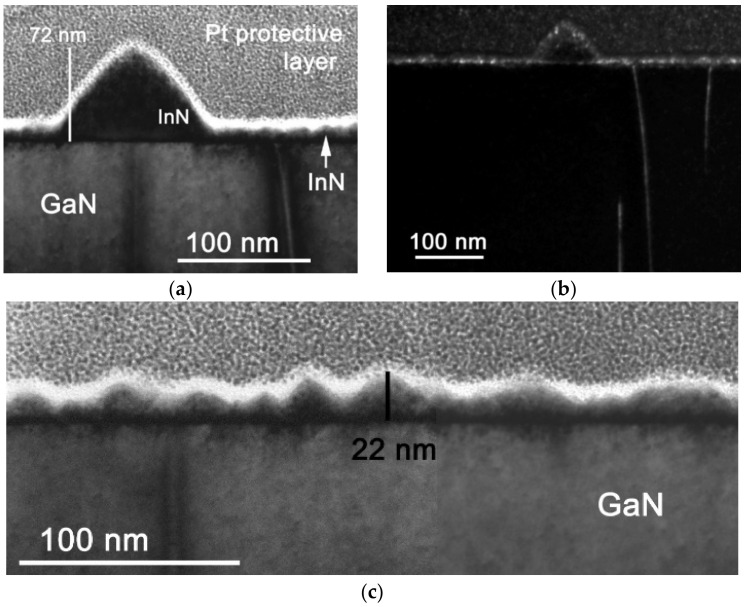
TEM cross-sectional views of the on-axis sample along the $\left[\bar{1}2\bar{1}0\right]$ InN direction. Bright field (BF) image of a large pyramid (**a**), weak-beam dark field (DF) image (**b**) with ***g*** = 0002 from the same area as (**a**), and BF of the continuous layer consisting of small pyramids (**c**).

SEM and TEM observation revealed that the In(Ga)N material consisted of a continuous layer built of homogeneously distributed small and large pyramids. (Figure 1, Figure 2 and Figure S1 in the Supplementary Material). The pyramids have conical shape with a slightly distorted hexagonal footprint reflecting the GaN and InN lattice symmetry. The conical shape preference is characteristic for InN dots grown at high deposition temperatures [20].

The AFM measurement showed that self-assembled growth of the large and small In(Ga)N pyramids was homogenous and resulted in bi-modal pyramid size distributions on both samples. This is exemplified with 5 × 5 μm^2^ scans in Figure S2 in the Supplementary Material. The root mean square (RMS) surface roughness (5 × 5 μm^2^) of both samples was 13 nm. When the large pyramids were excluded from the roughness analysis, RMS surface roughness (measured using smaller but comparable scans) dropped to ~2 nm and ~3 nm for the on-axis and off-axis sample, respectively. The slightly higher RMS surface roughness after the exclusion of the large pyramids was caused by patches of varying height, clearly visible on the surface of the off-axis sample and reflecting the stepped surface caused by the vicinal substrate (Figure 1b and Figure S1b). Due to the lack of this waviness, the surface of the on-axis sample was smoother.

**Figure 3:**
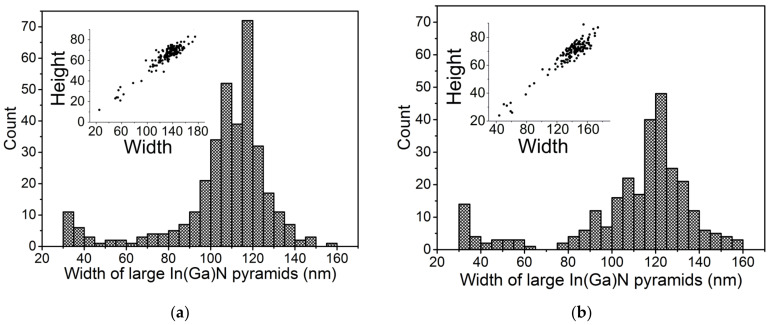
In(Ga)N pyramid width distribution on on-axis (**a**) and off-axis (**b**) samples. Insets show the correlation of their width and height.

Thorough SEM examination confirmed the bimodal distributions of pyramid dimensions (Figure 3). The width of a pyramid was determined as the length of a diagonal between two opposite vertices of its base hexagon. The layers were densely populated with pyramids whose lateral size varied between ~2 nm and ~180 nm. Most of the surface was covered in minute pyramids less than 10 nm across. Due to the coalescence of small pyramids, it was problematic to measure their widths, thus Figure 3 summarized the numbers of pyramids with the widths larger than 20 nm. The data was collected from twelve 2.071 × 1.380 mm^2^ images that were taken at various locations on the samples. Both on-axis and off-axis distributions revealed a similar trend: with the width decreasing from ~160 nm towards lower values, the count reached at first a local maximum, then a local minimum, and then it started to increase again.

The correlation between pyramid height and width revealed their aspect ratio of about 0.5 (see the insets in Figure 3a,b). The large pyramids grown on the on-axis substrate showed slightly narrower distribution of their height. Concentrations of large pyramids with widths between 30 and 160 nm were 10.3 × 10^8^ cm^−2^ on the on-axis substrate, and 8.2 × 10^8^ cm^−2^ on the off-axis substrate.

The most significant difference between the on- and off-axis samples was the presence of cracks on the off-axis oriented sample (Figure 1b,d). These were oriented mainly perpendicularly to the inclined surface steps. Because the cracks were narrow (see AFM scan and line profile in Figure S3 in the Supplementary Material), it was not possible to measure their depth. However, we suppose that they originated from GaN template stack. Similar oriented cracks appeared in GaN layers grown on off-axis substrates of 4H-SiC with different miscuts due to different mechanism of growth and strain relieving of GaN grown on stepped vicinal surfaces in comparison to one on-axis (0001) substrates [21,22]. Some clear steps are labelled by the asterisk and a crack by the thick short arrow in Figure 1b.

The large pyramid shape analysis showed that their side surfaces were not planar and their inclination changed along the pyramid height. While at the bottom of the pyramids the side surfaces followed the facets related to the $\left\{1\bar{1}01\right\}$ lattice planes, their top sides followed facets related to the $\left\{1\bar{1}02\right\}$ lattice planes (see Figure S4 in the Supplementary Material). According to Ploch et al., the $\left\{1\bar{1}02\right\}$-related facets are more frequent for InN pyramids as they are more stable at higher temperatures [20]. However, Bi et al. showed that $\left\{1\bar{1}01\right\}$-related facets were characteristic for In_0_._2_Ga_0.8_N hexagonal pyramids [23]. The observed change in the inclination angle of the large pyramid side walls suggests that as the pyramid grew, the molar fraction of Ga incorporated in their lattice (see the text below) diminished with increasing distance from the GaN buffer layer surface. The incorporation of Ga in the small and large pyramids can be accounted for considering Ga out-diffusion and/or a memory effect during the deposition of InN.

The whole In(Ga)N layer—the small and large pyramids—are epitaxially grown on GaN according to the relationship In(Ga)N $\left(0001\right)\;\left[10\bar{1}0\right]$ || GaN $\left(0001\right)\;\left[10\bar{1}0\right]$ || Al_2_O_3_ $\left(0001\right)\;\left[2\bar{11}0\right]$. The relationship was confirmed by electron diffraction (Figure S5 of the Supplementary Material) and the XRD analysis.

**Figure 4:**
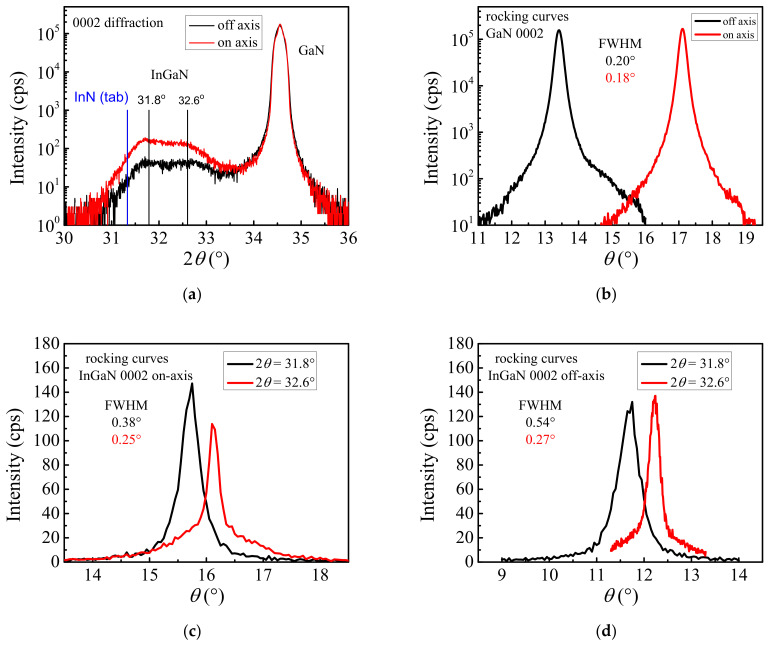
XRD *2θ/ω*-scan showing diffractions 0002 of GaN and In(Ga)N (**a**), corresponding rocking curves for GaN on the on-axis and off-axis samples (**b**) and rocking curves for two 0002 maxima of In(Ga)N measured on the on-axis (**c**) and off-axis (**d**) samples.

The 2*θ*/*ω* scans showing only the 0002 diffractions of GaN and In(Ga)N (as follows from the epitaxial relation) for the on-axis and off-axis samples are presented in Figure 4. The In(Ga)N 2*θ* maxima are slightly shifted to larger values compared to the tabulated value of 31.330° for InN (PDF 00-050-1239), indicating smaller lattice parameters. This is probably due to the incorporation of Ga atoms into the InN lattice. The rocking curves (*ω*-scans) measured at the positions of two local maxima of wide In(Ga)N 0002 diffractions showed a different misorientation of zones with different Ga molar fraction. As the pyramids had different heights we assume that the larger pyramids or at least their upper parts, where their side walls showed the inclination angle of 42.9°, contained less Ga. We expect the two observed In(Ga)N maxima in the symmetric XRD scans may correspond mainly to the large (2θ = 31.8°) and the small (2θ = 32.6°) pyramids and reflect their lower and higher Ga incorporation. A larger misorientation of the large pyramids (FWHMs of 0002 In(Ga)N ω-scans: 0.54° and 0.38° for off-axis and on-axis samples, respectively) then follows from their larger lattice misfit to GaN, probably accommodated continuously as the ratio of incorporated Ga decreased. However, the relatively narrow In(Ga)N *ω*-scan FWHM compared to that of GaN buffer reflects the good epitaxial orientation of In(Ga)N pyramids. The larger FWHM of 0002 In(Ga)N ω-scan for off-axis sample in comparison to the on-axis one is caused by the growth of large pyramids on the wavy surface of off-axis GaN (Figure 1b). We want to note that the all ω-scans were measured with the sample orientation where the axe of sample rotation was perpendicular to direction of miscut of the off-axis sample.

The lattice parameters, Ga molar fraction and strain states were calculated from the precise positions of the 0004 and $11\bar{2}4$ diffraction spots in the reciprocal space. While the 0002 In(Ga)N diffractions showed two distinguished maxima, the corresponding 0004 and $11\bar{2}4$ diffractions had much smaller intensities and only one clearly visible maximum belonging to material volume with higher lattice parameters. Thus the mentioned parameters were calculated for the In(Ga)N material with a smaller molar fraction ratio of Ga for each sample. However, due to weak and wide maxima of 0004 and especially $11\bar{2}4$ diffractions and due to presence of continuous gradient of Ga ratio in the layer the error of elemental cell volume estimation should be relatively high. The measurement procedure and calculation were described in detail elsewhere [24].

Obviously, in continuous epitaxial layers, the misorientation is expressed by densities of threading dislocations. The density of dislocations with screw (*N_disS_*) and edge components (*N_disE_*) was evaluated according to a model describing the *hkil* dependence of the dislocation induced broadening of the X-ray rocking curves [25]. One symmetric—0002—and one skew—$10\bar{1}1$—diffractions were measured and the FWHM of the third in-plane diffraction $10\bar{1}0$ was extrapolated. A random distribution of threading dislocations was assumed and their densities $N_{dis}$ were calculated as $N_{disS}=\frac{\Gamma_{0{}^{\circ}}^{2}}{4.35b_{s}^{2}}$ and $N_{disE}=\frac{\Gamma_{90{}^{\circ}}^{2}}{4.35b_{e}^{2}}$ [26]. $\Gamma_{\chi }$ are the FWHMs of rocking curves at the inclination angle χ=0° (diffraction 0002) and at the extrapolated value χ=90° (diffraction $10\bar{1}0$). $b_{s}$ and $b_{e}$ are the magnitudes of Burgers vectors of the screw and edge dislocations, respectively.

The calculated threading dislocation densities have a theoretical meaning in non-continuous layers. In fact, the pyramid misorientation was realized mainly by misfit dislocations at the pyramid/GaN interface and because of the 3D growth of pyramids with free-standing faceted side walls, no threading dislocations were necessary to accommodate their mutual lattice misorientation. Indeed, TEM observations showed that the volumes of large individual pyramids were free of threading dislocations. In some cases, when pyramids were touching each to other, threading dislocations penetrated the large pyramids (discussed below). However, the density of such dislocations was too small compared to misorientations determined by the XRD measurement.

The resulting data are summarized in Table 1. They show that the In(Ga)N layers were relaxed, an expected outcome for the InN dots grown on GaN, where the interface strain is relaxed very quickly during the early stage of growth due to the generation of a 60° misfit dislocation network [7].

The Ga incorporation ratios in In(Ga)N extracted from the positions of In(Ga)N diffractions corresponding to the pyramids with lower Ga molar ration were rather significant, between 15% to 20%, and lower in the on-axis sample than in the off-axis one, even if considering the low accuracy of their estimation due to weak intensities and wide maxima of used diffractions.

The different calculated dislocation densities reflect different character of the lattice misorientation for each sample. The on-axis sample exhibited larger twisting (rotation about the substrate normal) and a less significant tilting (misorientation from the [0001] orientation). This corresponds to the basic idea that the presence of oriented steps in the GaN buffer layer served as nucleation centres for the growth of In(Ga)N pyramids and could diminish their twisting. However, the steps with different local density and height could emphasize the In(Ga)N pyramid tilting.

The lack or presence of threading dislocations in the large pyramids was observed by TEM. The large pyramids were grown on the top of edge-type threading dislocations originating in the GaN layer (Figure 2a,b), but the dislocations did not penetrate them. In some cases, where the TEM lamellae did not cut through the centre of a large pyramid, the dislocation contrast in GaN was weak or missing.

The edge-character of dislocations was confirmed by observing the contrast of the strained lattice in the vicinity of a dislocation. The contrast is missing (or residual) if sample is oriented in such a way that the invisibility criterion for dislocations ***g.b*** = 0 is satisfied. There, ***g*** is the operating reflection and ***b*** is the dislocation Burgers vector [27]. Table 2 summarizes the important operating reflections used in this publication for the analysis of threading dislocations in GaN and InN.

**Figure 5:**
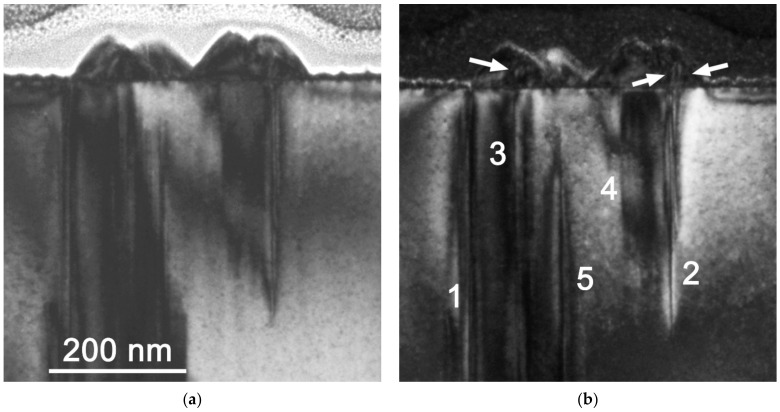

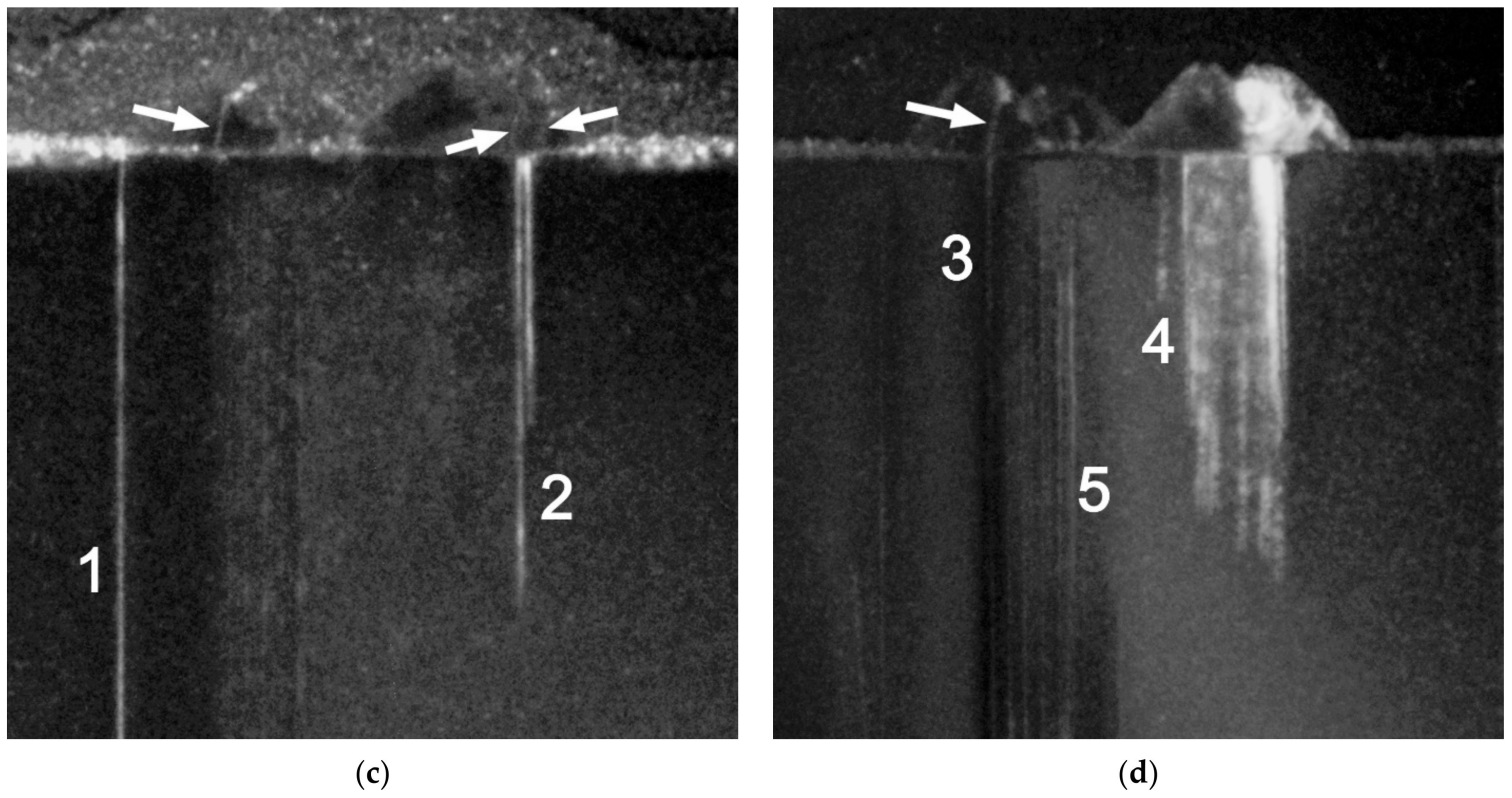
Cross-sectional TEM images along the $\left[\bar{1}2\bar{1}0\right]$ InN direction. Dislocation contrast variation is shown in BF (**a**) and DF (**b**) image along the zone axis *B* = $\left[\bar{1}2\bar{1}0\right]$ and weak-beam DF images using ***g*** = 0004 (**c**) and ***g*** = $10\bar{1}0$ (**d**).

Although the edge-type threading dislocations did not penetrate the large, isolated pyramids, the situation was different for pyramids that merged into larger clusters. Figure 5 illustrates this situation with some threading dislocations (see the arrows) that penetrated such coalesced pyramids.

**Figure 6:**
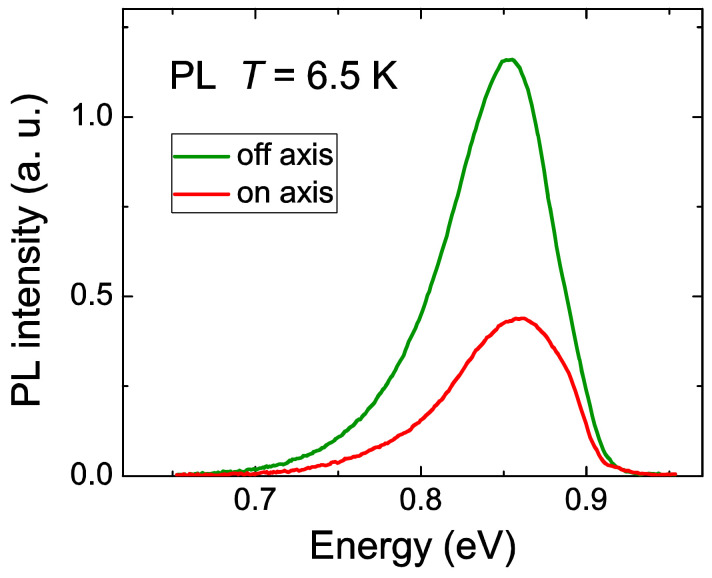
PL spectra of on-axis (lower intensity) and off-axis (higher intensity) samples measured at 6.5 K.

Figure 6 shows the PL spectra of the on-axis and off-axis samples measured at 6.5 K. The spectra exhibit a shape which is typical for heavy-doped semiconductors with the band-filling effect [28]. There is almost no difference between the PL line shapes, reflecting that the recombination processes and overall electronic system were similar in both samples. As the low energy tails of the spectra are positioned above the bandgap energy of the pure InN (0.69 eV at 0 K [3]), the PL measurement supports the finding that Ga atoms were incorporated in the layers and formed an In_x_Ga_1-x_N alloy. The PL maxima were at 853 meV with a FWHM of 75 meV and at 859 meV with a FWHM of 80 meV for the off-axis and on-axis samples, respectively. The on-axis sample exhibited a lower intensity of PL radiation.

In the XRD experiments a difference of about 5% of Ga content between the on-axis and off-axis In_1-x_Ga_x_N layers was found (Table 1). In standard homogeneous samples this should lead to the band-gap difference in the order of 100 meV. Contrary to that, PL of both samples exhibits rather close peak energies. This seeming discrepancy could be explained by recombination kinetics in an inhomogeneous material with fluctuating band-gaps. Excited electrons and holes relax preferentially to the positions in the sample with the narrowest band-gap, which could be very similar in the both samples.

The PL parameters are very similar to the published results on the relation between PL spectra shapes and composition of In_1-x_Ga_x_N alloys. Moret et al. showed the broadening of the PL spectra measured at 2 K from about 60 meV in InN to about 90 meV in an In_1-x_Ga_x_N alloy with a Ga molar fraction of 30% [29]. Tuna et al. reported on the PL maximum at 810 meV in a In_1-x_Ga_x_N sample with an indium molar fraction of 83% [30]. However, Kazazis et al. presented that the In molar fraction of 80% led to a PL maximum of about 1.00 eV, a higher value than that measured in our samples [13]. They performed a detailed investigation of the PL and variable angle spectroscopic ellipsometry of their samples. According to the authors, higher observed values of *E_g_* could result from a high quality of their radio-frequency molecular-beam epitaxy (RF-MBE)-grown samples, leading to much narrower band-tails.

**Figure 7:**
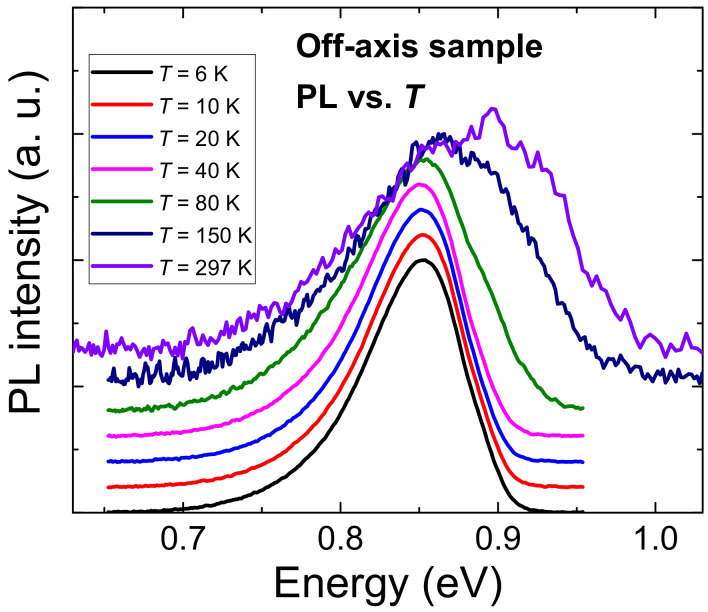
PL spectra of the off-axis sample in the temperature range from 6.5 K to the room temperature. The spectra are shifted in the *y*-axis for the purpose of a better view.

In Figure 7, PL spectra in the temperature range from 6.5 K to the room temperature are depicted. For the higher temperature range (from about 80 K to the room temperature) an unusually large shift of the maximum to higher energies could be observed. The shift could be explained by the increasing thermal energy of excited electron-hole pairs. At temperatures high enough, their thermal energy is sufficient to diffuse to the places with higher band-gap energies. This phenomenon, together with standard thermal broadening resulted in the blue-shift of the PL maximum, contrary to a standard red-shift caused by the band-gap thermal shrinking.

## 4. Discussion

The presented analyses suggest that the microstructure of both samples—on-axis and off-axis—was very similar with some interesting differences. As a result of self-assembled growth both samples consisted of a continuous thin layer of small In_1-x_Ga_x_N pyramids and randomly distributed large In_1-x_Ga_x_N pyramids with density of ~1 × 10^9^ cm^−2^. In both samples, symmetric (2θ/ω) XRD scans showed wide continuous double maxima related to In(Ga)N materials with different Ga molar fractions because the pyramids are relaxed, so the changes of c-parameter could not be caused by different strains. The calculated Ga incorporation into the InN lattice was higher for the off-axis sample, which can be ascribed to the smaller average height of the large pyramids if one expects that the Ga incorporation in In_1-x_Ga_x_N volume decreased with the increasing distance from the In_1-x_Ga_x_N/GaN interface indicated by the double maxima found in the XRD results and also by the bent side walls of the large pyramids. However, the accuracy of Ga incorporation estimation is low due to weak and wide maxima of measured diffractions.

It seems that presence of oriented cracks in GaN template grown on off-axis substrate indicating different mechanism of strain accommodation in GaN in this case did not influence significantly growth and properties of large In(Ga)N pyramids.

The TEM observations showed that large pyramids are grown over the edge threading dislocation. Their relationship could be explained by mechanism of self-assembled growth. As we have mentioned in the introduction, small island formation appeared very soon at the beginning of the growth to reduce the compressive strain energy due to the high misfit to the substrate. The islands were preferentially created on threading dislocations or surface steps—zones with locally decreased compression strain where larger In atoms can be preferentially located [31,32,33,34]. Such zones could act as constant zones of preferential InN or In(Ga)N growth and in this way support self-assembled growth of dots or pyramids with bimodal size distribution. This was supported by an observation of Kim et al., saying that the local maxima of In ratio in In-rich In(Ga)N layer consisted of small and large pyramids were found in zones of large pyramids [8]. Moustakas et al. grew about 15 nm high InN pyramids on GaN and showed that almost all pyramids nucleated above threading dislocations in GaN buffer [34]. They also presented a bimodal GaN pyramidal quantum dot distribution for GaN on AlN and showed correlation of larger dots growth with threading dislocations with edge components located in AlN. The fact that only In-containing precursor is constantly added into the growing chamber could increase the size difference between large and small pyramids in comparison to In_1-x_Ga_x_N deposition.

The large pyramids were well crystallized and mainly free of dislocations. The strain was relieved by the creation of 60° misfit dislocation networks on the island/GaN interfaces as it is obvious for hetero-epitaxial systems with a high misfit [7], thus the treading dislocations did not penetrate into the relaxed island. However, we observed threading dislocations passing the pyramids which coalesced with other ones. This can be explained by the presence of small threading dislocations in pyramid borders published by Lozano et al. [7,31]. They visualized the network of 60° misfit dislocations using plan-view TEM and showed that the misfit dislocations located on InN/GaN interface could be bend near the pyrmid edge and passed the thin part of the pyramid creating a system of very small threading dislocations around the pyramid. We were not able to distinguish such dislocations due to the presence of continuous ayer of small pyramids. When some pyramids coalesced, their touching area became the volume where two slightly differently misoriented lattices with their slightly different misfit dislocation networks and their edge threading dislocation systems interacted. As a result threading dislocations could penetrate the zone to relieve the strains.

We need to point out that the densities of dislocations calculated from FWHM of ω-scans are only a theoretic expression of tilting and twisting misorientation of epitaxially grown pyramids here. The basic necessary conditions of homogeneous distribution of threading dislocations in the layer and the fact that the lattice misorientations are realized only (or mainly) by this threading dislocation system are not fulfilled. We used this concept because it is widely used, sometimes without any control if the supposed conditions are satisfied. Thus it could be sometimes confusing.

The strongly coalesced small pyramids with the higher molar fraction of gallium were much more defective as the large ones. A possible reason that the PL spectra did not show any maxima splitting caused by the In(Ga)N crystals with two different Ga molar fractions was the presence of large quantity of recombination centres in the thin layer consisting of the small pyramids, effectively suppressing their PL emission. In such case, the PL emission from the on-axis sample should be stronger than that from the off-axis sample because of the higher total volume of on-axis large pyramids (the on-axis large pyramids had the slightly average higher density and height). Surprisingly, our observation was the opposite and the reason is not quite clear. The explanation needs further investigation, i.e., a correlation between the microstructure and In_1-x_Ga_x_N lattice quality and the PL spectra.

## 5. Conclusions

Using only NH_3_ and trimethylindium (TMIn) as the respective N and In precursors we deposited In(Ga)N layers with unintentionally incorporated Ga in InN lattice. The In(Ga)N layers consisted of a thin (~10–25 nm) continuous layer of small conical pyramids in which large conical pyramids with the approximate height of 50–80 nm were randomly distributed. The Ga incorporation were inhomogeneous, the Ga ratio were higher in the thin layer of small pyramids, and further, the Ga incorporation gradient was observed in large pyramids. The large pyramids were grown above the edge-type dislocations which originated in the GaN buffer; the dislocations did not penetrate the large, isolated pyramids. The large pyramids were well crystallized and relaxed with only a small quantity of defects, such as dislocations located at the contact zones of coalesced pyramids. The low temperature (6.5 K) photoluminescence spectra showed one clear maximum at 853 meV with a full width at half maximum (FWHM) of 75 meV and 859 meV with a FWHM of 80 meV for the off-axis and on-axis samples, respectively. It can be explained by suppressing PL emission from the thin layer consisting of the small pyramids due to presence of large quantity of recombination centres and by recombination kinetics in an inhomogeneous material with fluctuating band-gaps. Excited electrons and holes relax preferentially to the positions in the sample with the narrowest band-gap, which could be very similar in the both samples.

## Figures and Tables

**Table 1:** Ga molar ratio *x* in In_1-x_Ga_x_N, residual strain *ε_ll_* in In(Ga)N, density of screw and edge dislocations and FWHMs of 0002 rocking curves (*ω*-scans) calculated from the XRD measurements.

Substrate	Layer	*x* [%]	*ε_‖_* [%]	Screw d. [10^9^ cm^−2^]	Edge d. [10^9^ cm^−2^]	FWHM Δω [°]
off-axis	GaN	-	-	1.0	11.5	0.20
	In_1-x_Ga_x_N	20.2	−0.2	6.4	26.2	0.54/0.27 *
on-axis	GaN	-	-	0.8	37.4	0.18
	In_1-x_Ga_x_N	14.7	0.1	3.3	95.4	0.38/0.25 *

* The values are measured for two different maxima in 0002 In(Ga)N diffraction (see Figure 4c,d).

**Table 2:** Application of the invisibility criterion for threading dislocations in the wurzite GaN and InN structures; **g** is the operating reflection in TEM.

Dislocation Type	Burgers Vector	g=0001	$g=10\bar{1}0$
edge	1/3 $\langle 11\bar{2}0\rangle$	invisible	visible
screw	〈0001〉	visible	invisible
mixed	1/3 $\langle 11\bar{2}3\rangle$	visible	visible

## Data Availability

The data is available on reasonable request from the corresponding author.

## Supplementary Materials

The following supporting information can be downloaded at: https://www.mdpi.com/article/10.3390/nano12193496/s1, Figure S1. SEM images of In(Ga)N layers grown on the on-axis (a) and off-axis (b) substrates. Figure S2. 5 × 5 m^2^ AFM scans of on-axis (a,b) and off-axis (c,d) samples. Figure S3. AFM scan of a zone with oriented cracks (a) and AFM line profile through the longer crack (b). Figure S4. Cross-sectional TEM view of a large In(Ga)N pyramid along $\left[\bar{1}2\bar{1}0\right]$ InN direction. Figure S5. TEM cross-sectional SAED patterns of the interface between GaN and Al_2_O_3_ substrate (a) and In/GaN interface (b).
