# Supplementary material for: In(Ga)N 3D Growth on GaN-Buffered On-Axis and Off-Axis (0001) Sapphire Substrates by MOCVD
Source: Nanomaterials (Basel). 2022 Oct 6;12(19):3496. doi: 10.3390/nano12193496 (PMC9565245; doi:10.3390/nano12193496)
Supplement: Supplementary file 1 [file nanomaterials-12-03496-s001.zip › nanomaterials-1932137-supplementary.pdf]

Supplementary Material for

In(Ga)N 3D Growth on GaN-Buffered On-Axis and Off-Axis (0001) Sapphire Substrates by MOCVD

Alica Rosová *, Edmund Dobročka, Peter Eliáš, Stanislav Hasenöhrl, Michal Kučera, Filip Gucmann and Ján Kuzmík

Institute of Electrical Engineering, Slovak Academy of Sciences, Dúbravská cesta 9, 841 04 Bratislava, Slovakia

* Correspondence: alica.rosova@savba.sk

A. Large-scale SEM images

Large-scale SEM images show that the large pyramids are homogeneously distributed across the whole surface of the samples. The surface wavy character of the off-axis sample is well visible on such plan-view SEM images, too.

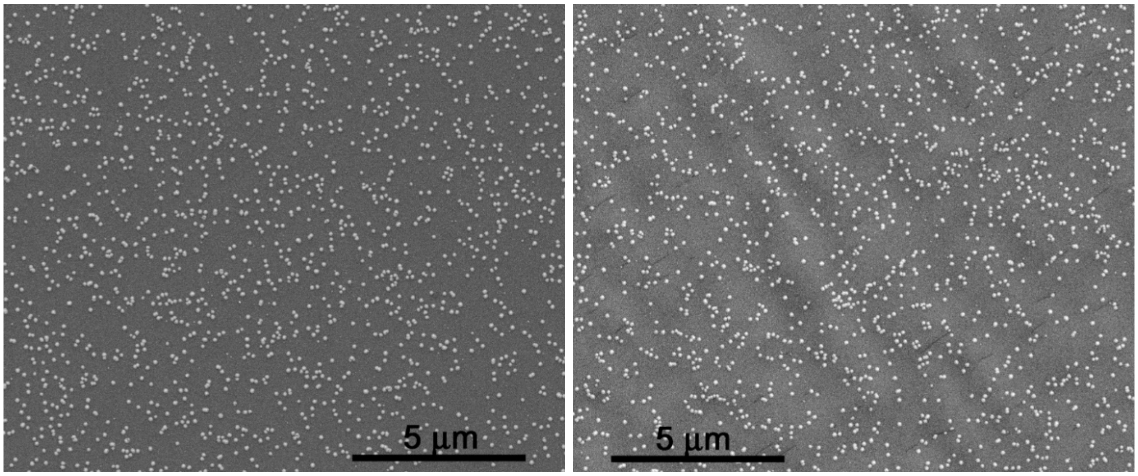

(a) (b)

Figure S1. SEM images of In(Ga)N layers grown on the on-axis (a) and off-axis (b) substrates.

B. AFM measurements

The homogeneity of In(Ga)N pyramids size distribution across the samples was verified by the AFM measurement. Figure S2 shows typical examples of 5 × 5 µm² scans of on-axis and off-axis samples.

Figure S3 represents an AFM scan of a zone on the off-axis In(Ga)N sample with cracks and a line-profile taken from the larger crack. The used AFM probe tip half angle is close to 20°, but may be lower for the first ~200nm of probe length. Because the crack is narrow, the depth of the crack is inconclusive, it may be deeper than showed or the crack may be transformed into very narrow trench at the bottom of measured crack profile and thus not resolved. However, we suppose the crack originated from strain accommodation of GaN template stack.

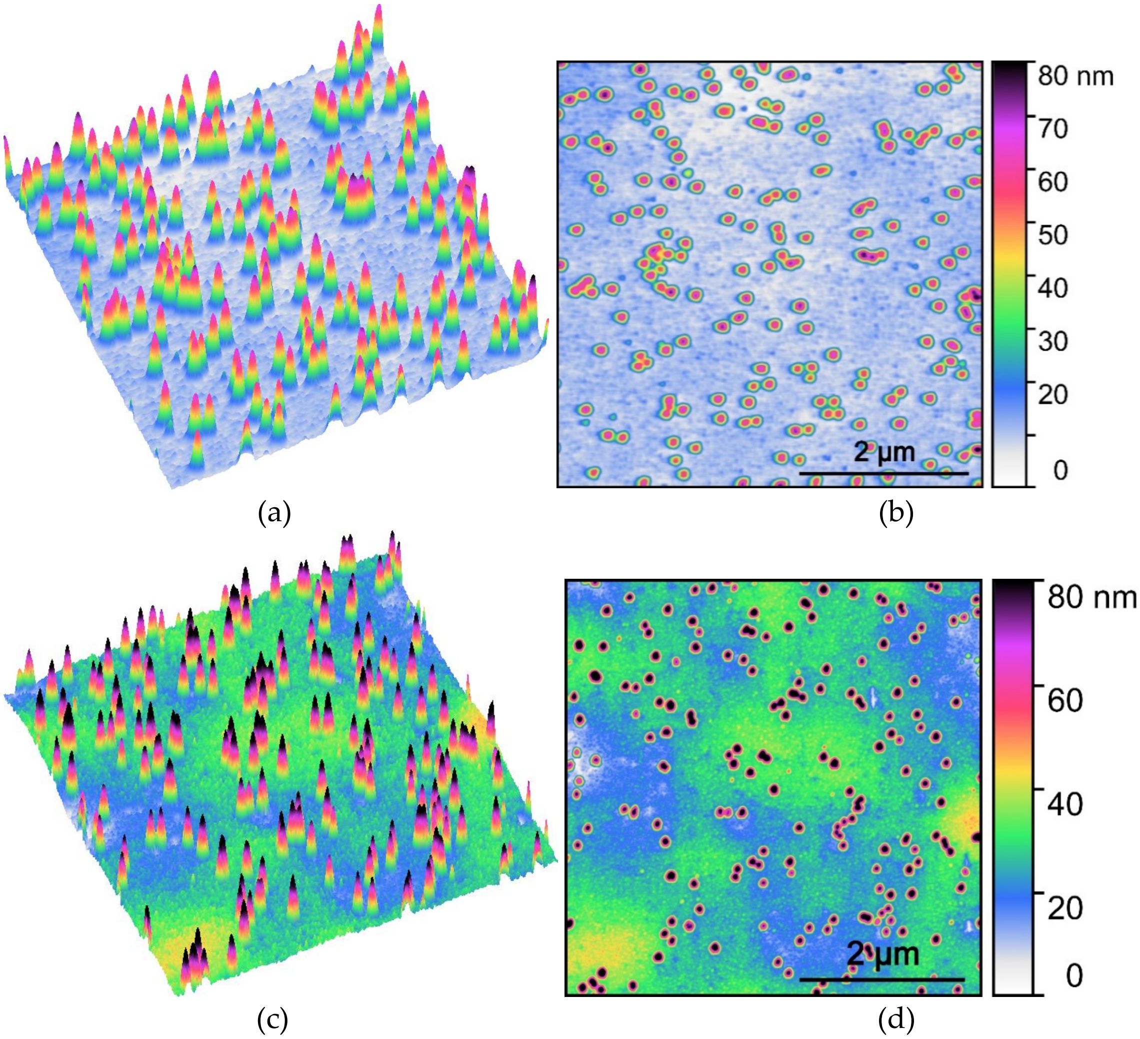

Figure S2. 5 × 5 µm^2 AFM scans of on-axis (a, b) and off-axis (c, d) samples.

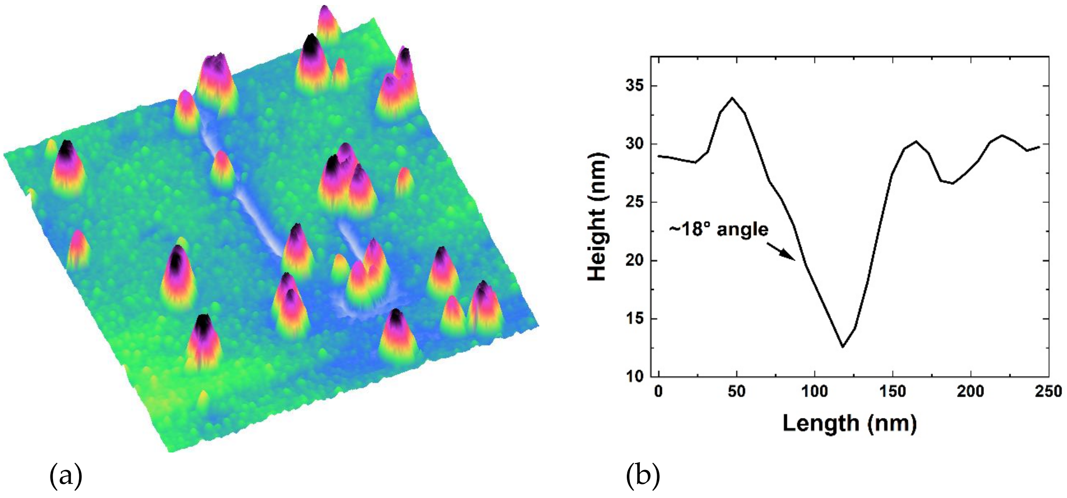

Figure S3. AFM scan of a zone with oriented cracks (a) and AFM line profile through the longer crack (b).

C. TEM analysis

TEM cross-sectional view of an individual large pyramid along $[\bar{1}2\bar{1}0]$ InN direction showing pyramid side walls is presented in the Figure S4. Instead of being completely straight, the side walls outline had slightly concave shape. The coloured lines show theoretic inclination of $(10\bar{1}1)$ lattice planes (the red line, inclined by 61.8° from the (0001) surface lattice plane) and $(10\bar{1}2)$ (the green line, inclined by 42.9° from the (0001) surface lattice plane).

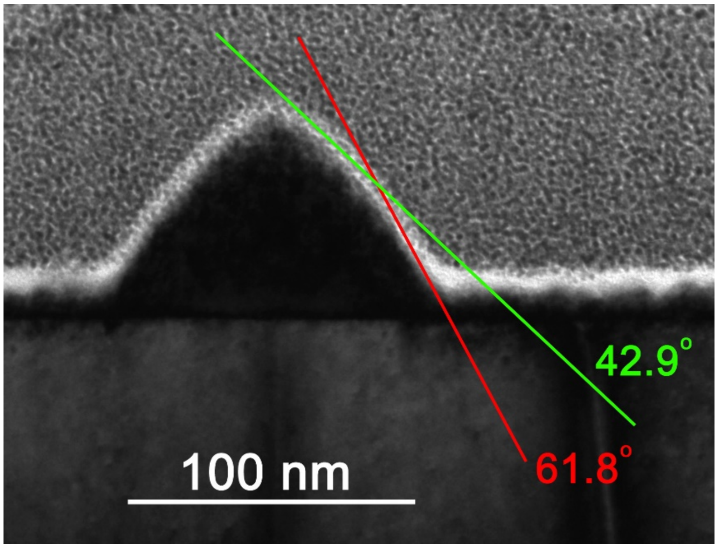

Figure S4. Cross-sectional TEM view of a large In(Ga)N pyramid along $[\bar{1}2\bar{1}0]$ InN direction.

The following epitaxial relationship was found: InN (0001) $[10\bar{1}0]$ || GaN (0001) $[10\bar{1}0]$ || Al_2O_3 (0001) $[2\bar{1}\bar{1}0]$ by electron diffraction patterns from the cross-sectional TEM (Figure S5).

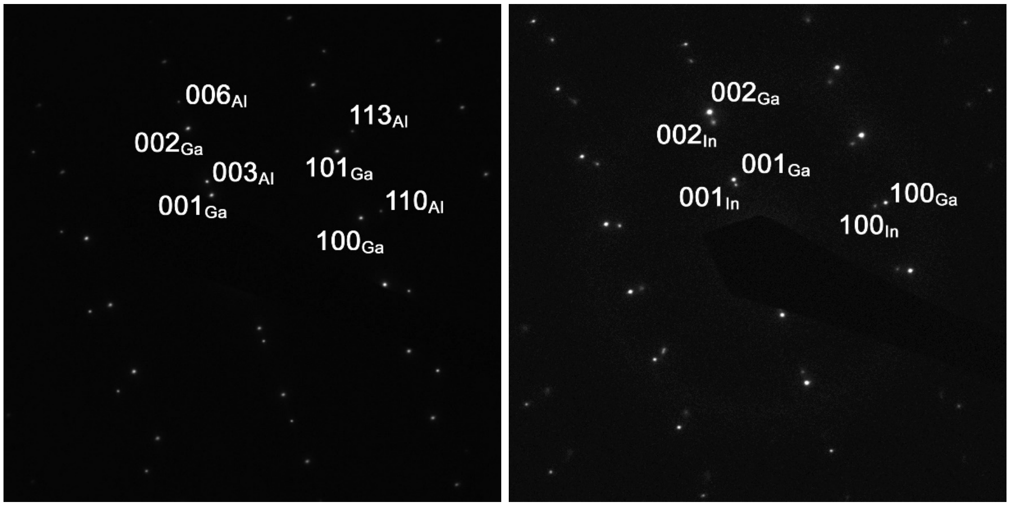

Figure S5. TEM cross-sectional SAED patterns of the interface between GaN and Al_2O_3 substrate (a) and In/GaN interface (b).
